# Model-Predicted Balance Between Neural Excitation and Inhibition Was Maintained Despite of Age-Related Decline in Sensory Evoked Local Field Potential in Rat Barrel Cortex

**DOI:** 10.3389/fnsys.2020.00024

**Published:** 2020-05-13

**Authors:** Sungmin Kang, Yurie Hayashi, Michael Bruyns-Haylett, Evangelos Delivopoulos, Ying Zheng

**Affiliations:** ^1^Biomedical Engineering, School of Biological Sciences, University of Reading, Reading, United Kingdom; ^2^Centre for Integrative Neuroscience and Neurodynamics (CINN), University of Reading, Reading, United Kingdom; ^3^Department of Bioengineering, Imperial College, South Kensington Campus, London, United Kingdom

**Keywords:** aging, excitation, inhibition, balance, field potential, modeling

## Abstract

The balance between neural excitation and inhibition has been shown to be crucial for normal brain function. However, it is unclear whether this balance is maintained through healthy aging. This study investigated the effect of aging on the temporal dynamics of the somatosensory evoked local field potential (LFP) in rats and tested the hypothesis that excitatory and inhibitory post-synaptic activities remain balanced during the aging process. The LFP signal was obtained from the barrel cortex of three different age groups of anesthetized rats (pre-adolescence: 4–6 weeks, young adult: 2–3 months, middle-aged adult: 10–20 months) under whisker pad stimulation. To confirm our previous finding that the initial segment of the evoked LFP was solely associated with excitatory post-synaptic activity, we micro-injected gabazine into the barrel cortex to block inhibition while LFP was collected continuously under the same stimulus condition. As expected, the initial slope of the evoked LFP in the granular layer was unaffected by gabazine injection. We subsequently estimated the excitatory and inhibitory post-synaptic activities through a balanced model of the LFP with delayed inhibition as an explicit constraint, and calculated the amplitude ratio of inhibition to excitation. We found an age-dependent slowing of the temporal dynamics in the somatosensory-evoked post-synaptic activity, as well as a significant age-related decrease in the amplitude of the excitatory component and a decreasing trend in the amplitude of the inhibitory component. Furthermore, the delay of inhibition with respect to excitation was significantly increased with age, but the amplitude ratio was maintained. Our findings suggest that aging reduces the amplitude of neural responses, but the balance between sensory evoked excitatory and inhibitory post-synaptic activities is maintained to support normal brain function during healthy aging. Further whole cell patch clamp experiments will be needed to confirm or refute these findings by measuring sensory evoked synaptic excitatory and inhibitory activities *in vivo* during the normal aging process.

## Introduction

Sensory evoked cortical field potentials have been shown to change during healthy aging (Onofrj et al., 2001; Wong, 2002). The characteristics of the changes vary depending on a range of factors such as cortical regions where neural signals were recorded, and types of stimuli. Many studies demonstrated age-related slowing of sensory evoked potentials, manifested as an age-related decrease in the peak amplitude of the evoked potential (Parthasarathy et al., 2014, 2019; Missonnier et al., 2004), or an increase in peak latency (Tremblay et al., 2004; Rufener et al., 2014; Gupta and Gupta, 2016), or both (Konrad-Martin et al., 2012; Brown et al., 2019). However there are also studies showing the opposite effect, with an age-related increase in the peak amplitude of the evoked potential (Sterr and Dean, 2008) or a decrease in peak latency with age (Herrmann et al., 2017).

To understand the mechanisms underlying these changes, we investigated how aging may alter sensory evoked post-synaptic activity which is the primary source of extracellular field potential recordings in the neocortex. The neural activity used in this study is the local field potential (LFP) which is the lower frequency component (<500 Hz) of the extracellular field potential recorded in the neocortex. LFP is closely associated with the excitatory and inhibitory post-synaptic currents (EPSC and IPSC) of the local pyramidal neural population (Logothetis and Wandell, 2004; Buzsáki et al., 2012; Einevoll et al., 2013; Haider et al., 2016). At the level of single neurons, these currents have been shown to co-vary, i.e., stronger or weaker excitation is accompanied by stronger or weaker inhibition, with inhibition lagging excitation by a few milliseconds (Wehr and Zador, 2003; Zhang et al., 2003; Higley and Contreras, 2006; Okun and Lampl, 2008; Atallah and Scanziani, 2009; Dehghani et al., 2016). This temporal delay of inhibition provides a brief time interval during which the membrane potential of a neuron depolarizes above the threshold for action potential firing. This principle of co-tuning is referred to as the balance between neural excitation and inhibition.

The temporal dynamics of the LFP are also dependent on the depth of the recording microelectrode probe in the neocortex (Einevoll et al., 2013), making mathematical modeling of the LFP more complex. Most existing neural population models are based on known connections between excitatory and inhibitory neural populations and are optimized to replicate frequency domain characteristics of the LFP or the scalp electroencephalogram (EEG) during resting state (Jansen and Rit, 1995; Brunel and Wang, 2003; Alvarez and Destexhe, 2004; Bojak et al., 2010; Coombes, 2010). Although these neural models incorporated components of excitation and inhibition, the constraints on their co-tuning characteristics and the temporal delay of inhibition to excitation were rarely enforced when model parameters were optimized.

A phenomenological LFP model was proposed to explicitly exploit the above two constraints and was aimed at modeling evoked LFP responses in the granular layer of the neocortex (Zheng et al., 2012). It was demonstrated that the model could be used to fit somatosensory evoked LFP data, to estimate neural excitation and inhibition, and to examine their balance during sensory adaptation. Crucial to this model was the implementation of the delay of inhibition with respect to excitation, constraining the initial segment of the sensory evoked LFP to reflect neural excitation only. To validate this, further experiments were conducted to manipulate the balance between neural excitation and inhibition through pharmacological intervention (Bruyns-Haylett et al., 2017). This was done by micro-injecting the gamma-aminobutyric acid A (GABAa) receptor antagonist bicuculline into the barrel cortex of a rat to eliminate the IPSC locally and collecting neural data while the whisker pad of the rat was stimulated. It was found that the initial segment of the evoked LFP was unaltered by the intervention, confirming that this segment was independent of inhibition. This finding implied that changes of the evoked LFP within the initial time window can be used to infer changes in neural excitation.

The objectives of the current study were (i) to investigate the effect of healthy aging on the temporal dynamics of somatosensory evoked LFP in the rat barrel cortex; (ii) to further demonstrate that the initial segment of the evoked LFP solely reflected synaptic excitation for all age groups, and (iii) to estimate the excitatory and the inhibitory neural activities from LFP responses using the balanced model of the LFP, and to compare their ratio across age groups to assess the balance of neural excitation and inhibition during the aging process.

## Materials and Methods

All procedures were undertaken in accordance with the 1986 Animal (Scientific Procedures) Act, under approval from the United Kingdom Home Office and approved by the Research Ethic Committee at the University of Reading, United Kingdom.

### Animal Preparation and Surgery

Detailed surgical procedures were published previously (Kang et al., 2017). They are briefly reviewed below.

Female Lister hooded rats were used from three age groups: pre-adolescence (PA), young adult (YA) and middle-aged adult (MA). The age and weight ranges for the three groups are: PA: 4–6 weeks (91–115 g); YA: 2–3 months (210–250 g); and MA: 10–20 months (285–380 g). The MA group rats were retired breeders. At the time of purchase their age range was only guaranteed to be between 12 and 40 weeks by the supplier. They were kept in-house for 7–10 months before surgery, hence the wide range of their age. Rats were housed in a temperature controlled room with a 12-h dark:light cycle with ad libitim access to food and water.

On the day of surgery, the rat was weighed and then anesthetized in an induction chamber supplied with 5% isoflurane (ISO) and pure oxygen at a flow rate of 1 L/min. The animal was then moved to a stereotaxic frame with its head fixed with ear and tooth bars, while ISO was supplied through a nose cone at 3% with an oxygen flow rate of 0.5 L/min during surgery. The location of the barrel cortex was determined by the stereotaxic coordinates (Paxinos and Watson, 2005) and a small hole (1–2 mm in diameter) above the barrel cortex was drilled into the skull without damaging the dura. For the YA group, this location was at 6 mm laterally and 2.5 mm posteriorly from the bregma. As the size of the brain increases during aging (Morterá and Herculano-Houzel, 2012), the location of the hole was proportionally modified for the PA and the MA groups based on the distance between the bregma and the lambda. After the bottom of the hole was thinned to translucency, a needle was used to pierce the dura to allow the insertion of a micro-electrode.

The rat was then transferred to a Faraday cage mounted on top of a vibration isolation workstation. The hard-plastic nose cone for isoflurane administration, together with the bite bar, were then replaced with a microflex breather fitted with a transparent soft nose cone for isoflurane delivery. This was because the hard-plastic nose cone covered almost the entire whisker pad of the rat, making whisker pad stimulation very difficult. By switching to a soft nose cone, which was modified by cutting a semi-circle off the cone, it allowed easy whisker stimulation to one side of the whisker pad without compromising the isoflurane administration (Kang et al., 2017).

Simulation comprised of brief electric current pulses with an intensity of 1.2 mA applied to the right whisker pad using two tungsten electrodes. In the contralateral barrel cortex, a 16-channel recording micro-electrode (NeuroNexus Technologies) was used for extracellular field potential recordings. The tip of the electrode consists of a linear array of 16 recording sites, with inter-electrode spacing of 100 μm and area of each site 177 μm^2^. The electrode was placed above the burr hole perpendicularly to the pia surface, typically 20–30° to the vertical direction. It was advanced under visual (through a microscope) monitoring until just touching the pia surface of the burr hole. It was then inserted normal to the cortical surface to a depth of 1,500 μm, with the uppermost electrode site at the cortical surface.

During neural recording, physiological parameters of the rat was monitored via an oximeter control unit (MouseOxPlus, Starr Life Sciences Corp., World Precision Instruments, United Kingdom) by attaching an oximeter sensor clamp to the rat’s hind paw. ISO was varied between 0.7 and 1.2% to maintain the level of anesthesia at stage III3 (Friedberg et al., 1999; Devonshire et al., 2010), while O_2_ flow was kept at 0.4–0.5 L/min. The sensory evoked responses presented in this paper were averaged over 300 trials with an inter-trial interval (ITI) of 10 s. All neural recordings were sampled at 24.41 kHz. After each experiment, the rat was sacrificed, and the brain was extracted as quickly as possible. Total brain weight was recorded using a precision scale (0.01 g sensitivity).

### Anesthetic Level Monitoring

The modulatory effects of anesthesia on spontaneous and evoked neural activity have been demonstrated by several studies (Masamoto et al., 2009; Devonshire et al., 2010; Aksenov et al., 2015; Kortelainen et al., 2016). In order to compare neural responses across different groups under an anesthetic regime, it is important that the anesthetic levels for all groups are kept the same. We monitored the anesthetic level primarily by observing the temporal dynamics of extracellular field potential recordings shown on a PC monitor throughout the recording period. It is well-known that spontaneous neural signals display distinct temporal dynamic characteristics at different stages of anesthesia (Friedberg et al., 1999; Devonshire et al., 2010). At the deep anesthetic level IV, the spontaneous neural signal has little variation. As the anesthetic level is decreased to level III4, spindles begin to appear. These are self-sustained oscillations lasting from around 0.5–2 s, increasing in the frequency of occurrence as the percentage of ISO is decreased. When the field potential recording appears as a continuous fluctuation over time, stage III3 is reached. Further decrease in ISO concentration will reduce the anesthetic level to stage III2, resulting in a significant reduction in the amplitude fluctuation of the spontaneous field potential, accompanied by a significant increase in the animal’s heart and respiration rates. We actively prevented the anesthetic level to reach stage III2 or lighter, as these stages may induce eyelid reflex or whisker movement which were undesirable.

All neural data presented here were collected during stage III3. To maintain this level of anesthesia, the ISO concentration was carefully adjusted. If the anesthetic level was changed significantly during a recording session, the recording would be stopped, ISO re-adjusted and neural signal re-collected.

The anesthetic level was further assessed post-experiment by computing the power spectral density (PSD) of the spontaneous neural activity in the Gr layer, as it has been shown that at the anesthetic level III3, the resting state PSD should have a peak around 3–4 Hz (Friedberg et al., 1999). The PSD was computed using the Matlab function pwelch() and the spontaneous LFP was taken as 1–9 s after each stimulus onset.

### Pharmacological Manipulation of Neural Inhibition

In a recent study (Bruyns-Haylett et al., 2017), we infused bicuculline (GABAa receptor antagonist) into the barrel cortex of rats to block inhibitory synapses of local cortical neurons, and showed that the amplitude of the sensory evoked LFP was enhanced, while the initial slope was unchanged. We suggested that the initial segment of the LFP reflected only the excitatory post-synaptic potential because of the temporal lag of inhibition with respect to excitation. That study used urethane-anesthetized rats.

In this study we aimed to replicate our previous findings under a different anesthetic condition. Instead of urethane, we used the ISO anesthetic regime (Kang et al., 2017). Additionally, we infused a different GABAa receptor antagonist, gabazine, into the barrel cortex. To select the appropriate concentration of gabazine at the infusion rate of 0.2 μl/min for 2 min, a range of concentrations was tested in a pilot study: 15, 5, 3, and 1.5 μM (Sokal et al., 2000; Kernig et al., 2012). At the two higher concentrations, pronounced epileptiform activity was observed, whereas at 1.5 μM concentration, little drug effect was found. At the concentration of 3 μM, evoked LFP responses increased post drug infusion with limited epileptiform spikes present immediately after drug infusion. However, these spikes disappeared ∼5–10 min. After drug infusion, while evoked LFP responses were still elevated. Thus, gabazine with 3 μM concentration was chosen to be infused into layer VI of the barrel cortex via a 16-channel fluidic laminar microelectrode (NeuroNexus Technologies).

Another finding from the pilot study was that the effect of gabazine on the LFP lasted several hours, i.e., the amplitude of the evoked LFP was enhanced by gabazine infusion and only gradually returned to baseline after several hours. This period was much longer than the effect of bicuculline which lasted about 30 min. Therefore, the drug infusion experiment was conducted as the last run of the day, with an ITI of 5 s for 300 trials. The infusion protocol consisted of three periods: the pre-infusion period (0–250 s, 50 trials), the infusion period (250–375 s, 25 trials), and the post-infusion period (375–1,500 s, 225 trials). The data set was then divided into 6 epochs, with 50 trials per epoch. The average evoked LFP from the first epoch was taken as the baseline LFP profile. Epochs 2 and 3 were not used due to the presence of epileptiform spikes. The average evoked LFP response from epoch 4 was used as a representative for the post-infusion neural response.

### Data Pre-processing and Parameter Estimation

Extracellular field potential data (sampled at 24.41 KHz) were pre-processed using our laboratory’s standard procedure (Bruyns-Haylett et al., 2017). Briefly, all data were aligned with stimulus onset. The stimulus artifact was removed, and the data were zero-meaned at the baseline and low pass filtered at 800 Hz. Inverse Current Source Density (spline iCSD, source radius *R* = 0; Pettersen et al., 2006) analysis was conducted to identify the layer IV sink channel, which has an early onset and a large negative peak (Mitzdorf, 1985) for each subject. The neural data were then aligned according to their granular layer sink locations (CSD data) across animals with the sink channel placed 600 μm (i.e., channel 7) below the pial surface. After re-alignment, the evoked LFP responses were calculated by averaging over 300 trials for each animal.

After pre-processing, four parameters associated with evoked LFP were extracted from individual trials to compare the dynamic characteristics of the evoked LFP responses across age groups:

• Peak amplitude: the maximum absolute value of the evoked LFP.
• Peak latency: the time the evoked peak amplitude is observed.
• Onset time: the time that 2% of the evoked peak amplitude is observed.
• Initial slope: the slope from 2 to 25% of the evoked peak amplitude.

For the drug infusion study, the same pre-processing procedure for sensory-evoked responses was applied. Some data sets were excluded due to large artifacts or noise in the data induced by drug infusion. Also, the evoked LFP data occasionally exhibited spiking activity during the epoch 4 period 10 min after gabazine injection. In these cases, trials were excluded from the analysis based on the following criteria: (i) An epileptiform spike was detected within a 1 s window before stimulus onset. (ii) If the standard deviation of the data 1 s before stimulation was > 5 × the standard deviation of the baseline (average over 50 trials). (iii) Data with greater than 20 contaminated trials were excluded. The post-infusion LFP (average from epoch 4) was then compared to the baseline LFP within each subject by normalizing the per subject LFP time series with respect to the mean baseline LFP peak.

### Balance of Synaptic Excitation and Inhibition Using a Mathematical Model

A mathematical model of the LFP (Zheng et al., 2012) was used to investigate whether the aging process changed the balance between neural excitation and inhibition. Seven model parameters were optimized (Appendix) using a non-linear least squares algorithm (Levenberg–Marquardt algorithm, Matlab function “lsqnonlin”) for each animal to fit the mean evoked LFP time series during the time period 0 ≤ *t* ≤ 200 ms. Excitatory and inhibitory components of the LFP were then estimated from the model using the optimized parameters. The balance between inhibition and excitation was calculated from the ratio.

(1) $$\rho =\frac{Peakampofthepredictedinhibitorycomponent}{Peakampofthepredictedexcitatorycomponent}$$

### Statistical Analysis

To compare parameters extracted from evoked LFP responses and estimated parameters from the LFP model across the three age groups, one-way ANOVA was performed for each parameter. If a significant difference was found, a *post-hoc* comparison was conducted using the Bonferroni correction by adjusting the corresponding *p*-value to the number of comparisons. Paired Student’s *t*-tests were used to compare the gabazine effect on the amplitude and the initial slope of evoked LFP responses pre- and post-infusion. To compare the ratio ρ across the three age groups, the non-parametric Kruskal-Wallis test was used. All parameters were expressed as the mean ± standard error (SE). Analyses were performed using the Matlab statistics toolbox. Significance was set at *p* < 0.05.

## Results

### Physical and Physiological Changes

**FIGURE 1:**
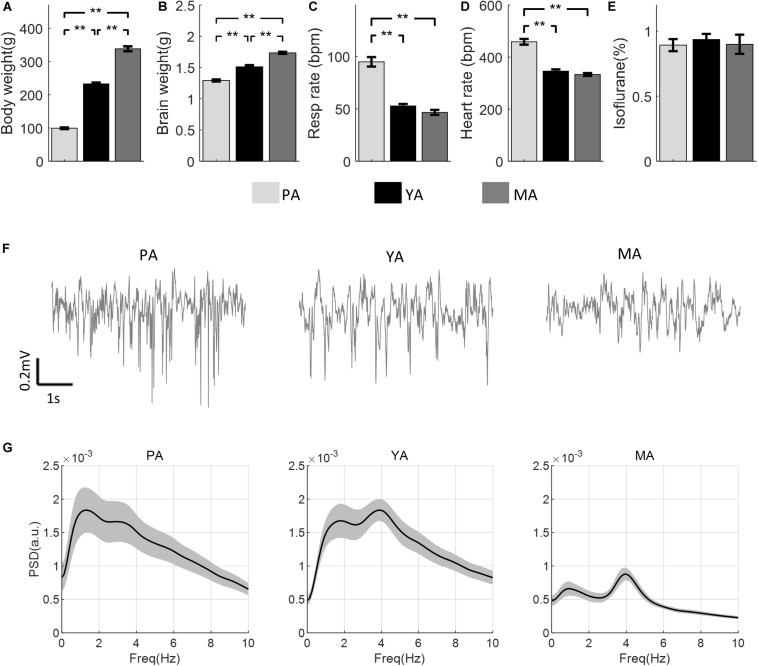
Comparisons of physical and physiological measurements across age groups (PA, YA, and MA). **(A)** Body weight. **(B)** Brain weight. **(C)** Respiration rate. **(D)** Heart rate. **(E)** ISO concentration. Error bars represent SE. Significance levels are represented with Bonferroni correction, **p < 0.003. **(F)** A representative field potential recording from the Gr layer during resting state from each age group. **(G)** PSD of the resting state LFP for each age group (PA, YA, and MA). Shadows indicate the SE of the PSD across subjects.

The mean body and brain weights of rats in each age group are shown in Figures 1A,B respectively. Both increased significantly with age [*F*(2, 30) = 444.23, *p* < 0.001 for body weight, *F*(2, 30) = 121.86, *p* < 0.001 for brain weight]. *Post-hoc* comparisons using Bonferroni correction indicated significant differences between all paired comparisons (*p* < 0.003) between groups. Figures 1C,D show respiration and heart rates (beat per minute, bpm) respectively for all age groups. There was a significant effect of age on both the respiration rate [*F*(2, 23) = 72.14, *p* < 0.001] and the heart rate [*F*(2, 23) = 70.92, *p* < 0.001]. *Post-hoc* comparisons using the Bonferroni correction indicated that mean respiration as well as heart rates in the PA group were significantly higher than those in the YA and the MA groups, but no significant difference in these parameters was found between the YA and the MA groups. In addition, no significant difference in the ISO concentration was found across age groups [*F*(2, 23) = 0.82, *p* = 0.2], as shown in Figure 1E. Representative resting state field potentials, one from each age group, are shown in Figure 1F. The mean PSDs of the resting state Gr LFP for the three age groups are shown in Figure 1G in the frequency range 0–10 Hz. Each PSD shows two local peaks, one around 1 Hz, the other between 3 and 4 Hz. The former was due to respiratory rate not well isolated in some experiments, while the later peak confirmed that the anesthetic level III3 was reached during data recording.

### Aging Reduced the Amplitude of the Evoked LFP While the Peak Latency Was Increased

**FIGURE 2:**
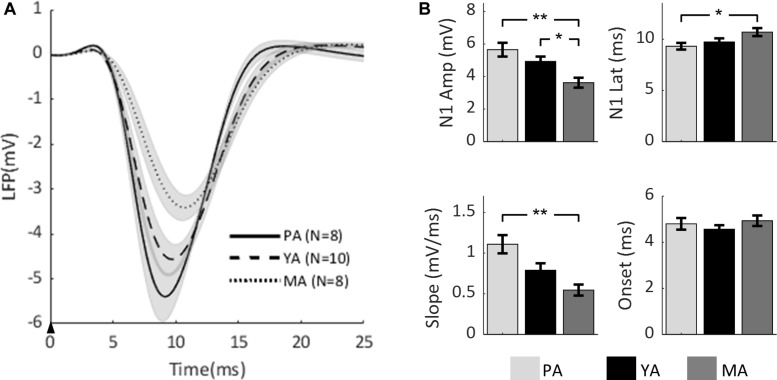
Comparison of evoked LFP across age groups. **(A)** Mean LFP of the PA, YA, and MA groups were superimposed. Shadows indicate the SE of responses across subjects. The black arrow indicates the onset of stimulus lasting 0.3 ms. **(B)** Bar plots showing statistical results of the LFP characteristics: N1 peak amplitude (top left), N1 peak latency (top right), initial slope (bottom left) and onset latency (bottom right) for PA, YA, and MA rats with error bars representing SE. Significant differences are represented with Bonferroni correction, *p < 0.017 and **p < 0.003.

To compare the effect of aging on the temporal dynamics of sensory-evoked LFP responses across age groups, the time series of LFPs in the Gr layer from all age groups are plotted in Figure 2A. Parameters characterizing the evoked LFP responses were extracted and are shown in Figure 2B. Peak amplitude of N1 (top left panel) was found to be significantly decreased with age [*F*(2, 23) = 8.53, *p* = 0.0017]. A *post-hoc* analysis showed that the N1 peak was significantly higher in the PA and YA groups compared with the MA group. With regard to N1 peak latency (top right panel), it was found to be significantly reduced with age [*F*(2, 23) = 3.7, *p* = 0.0406]. A *post-hoc* analysis indicated that this latency was significantly shorter in the younger group (PA) compared to the older groups. As to the initial slope of the evoked LFP (bottom left panel), aging significantly reduced this slope [*F*(2, 23) = 9.13, *p* = 0.0012]. A *post-hoc* analysis showed that the slope of the PA group was significantly higher than that of the MA group. However, in terms of the onset latency (bottom right panel), no significant effect of aging was found [*F*(2, 23) = 0.79, *p* = 0.4653]. These results suggest that both the strength and the temporal dynamics of the evoked neural response decreased with age.

### Gabazine Infusion Increased the Amplitude of the Evoked LFP but the Initial Slope Was Unchanged

**FIGURE 3:**
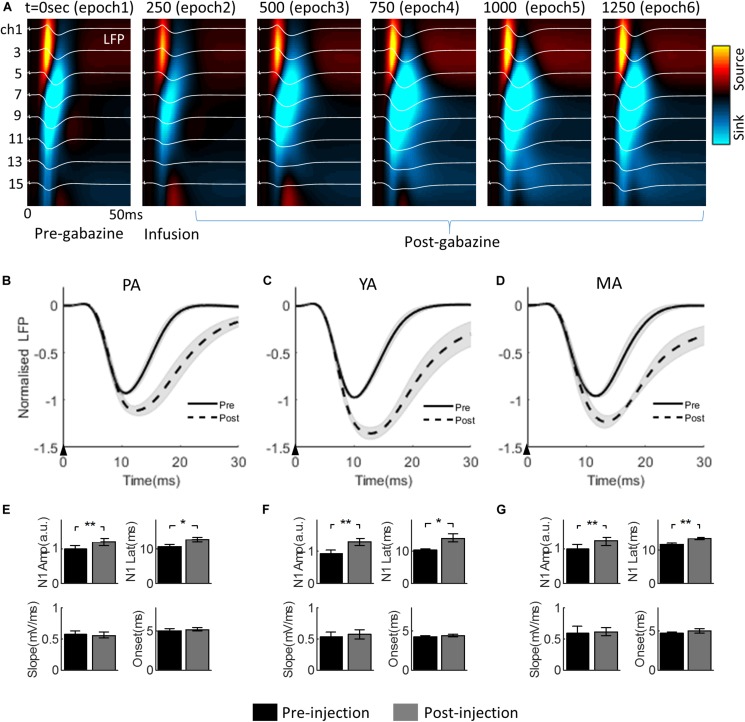
Effect of gabazine on the laminar LFP and CSD. **(A)** Progression of gabazine effect on the evoked LFPs (white curves) superimposed on the corresponding CSD maps. The PA group (*n* = 9) was displayed as a representative example. The x-axis: time from the stimulus onset to 50 ms; the y-axis: cortical depth 0–1,500μm (top to bottom) from the pia mater. Each subplot shows a group averaged response over 250 s period (i.e., 50 trials), with the starting time of the epoch period indicated at the top left of each subplot. **(B–D)** show gabazine effect on the temporal dynamics of the Gr layer LFP for PA, YA, and MA groups, respectively. LFPs pre- and post-gabazine injection are plotted as solid and broken curves, respectively. The black arrow in each panel indicates the onset of stimulus lasting 0.3 ms. **(E–G)** are bar plots of extracted parameters from evoked LFP responses pre- and post-injection for PA, YA, and MA groups respectively. Top left: N1 peak amplitude; top right: N1 peak latency; bottom left: initial slope of N1; and bottom right: onset latency. Error bars indicate SE. Asterisks indicate significant differences, *p < 0.05, **p < 0.01.

The progressive change of the evoked LFP associated with gabazine infusion is plotted in Figure 3A (for the PA group only), superimposed with the corresponding CSD maps. Infusion took place during epoch 2. The effect of gabazine infusion can be observed in epochs 4, 5, and 6 with enhanced and prolonged neural responses.

The Gr layer mean evoked LFPs during the baseline (epoch 1, solid curve) and the post drug infusion (epoch 4, dotted curve) are superimposed in Figure 3B (PA), Figure 3C (YA), and Figure 3D (MA) respectively. Parameters characterizing the dynamics of the evoked LFP pre- and post-infusion periods were extracted and are shown in Figure 3E (PA), Figure 3F (YA), and Figure 3G (MA). Gabazine infusion significantly changed the N1 peak amplitude and latency for all three age groups. However, both the initial slope and the onset latency of the N1 deflection were statistically unchanged (Table 1). These results replicated our previous findings using bicuculline with animals under a urethane anesthetic regime. They confirmed that the initial part of the somatosensory evoked LFP response is dependent only on the excitatory post-synaptic activity of the local pyramidal neural population.

**TABLE 1:** Mean ± SE of the differences in evoked LFP parameters pre- and post-gabazine infusion, and corresponding *p*-values in brackets (*p < 0.05, **p < 0.01).

	PA (m ± se)	YA (m ± se)	MA (m ± se)
N1 amplitude diff	0.17 ± 0.04 (0.0020) **	0.35 ± 0.04 (0.000) **	0.20 ± 0.03 (0.000) **
N1 peak latency diff (ms)	1.86 ± 0.64 (0.0194)*	3.77 ± 1.25 (0.0234)*	1.78 ± 0.41 (0.0049)**
N1 initial slope diff	0.02 ± 0.02 (0.4630)	0.03 ± 0.02 (0.0873)	0.02 ± 0.05 (0.7093)
N1 onset diff (ms)	0.16 ± 0.11 (0.1869)	0.11 ± 0.10 (0.3114)	0.36 ± 0.20 (0.1159)

### The Balance of Model Predicted Excitation and Inhibition Was Unchanged Across Age Groups

**FIGURE 4:**
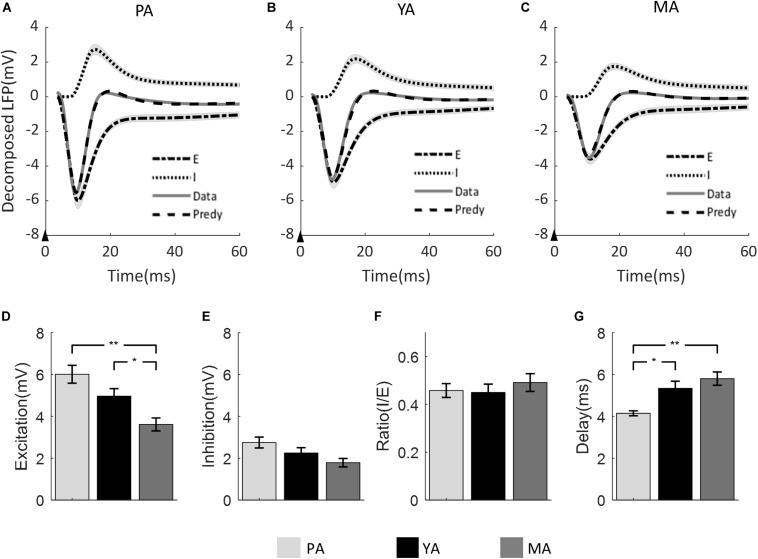
Model prediction across age groups. The measured mean LFP is superimposed with the model predicted LFP, and the model estimated excitatory and inhibitory components for **(A)** PA; **(B)** YA; and **(C)** MA groups. Shadows indicate the SE across subjects. The black arrow in each panel indicates the onset of stimulus lasting 0.3 ms. Bar plots show **(D)** the amplitude of excitatory component, **(E)** the amplitude of inhibitory component, **(F)** the ratio of inhibitory over excitatory amplitude, and **(G)** the delay of inhibition with respect to excitation across age groups. Error bars indicate SE. Significant differences are represented with Bonferroni correction, *p < 0.017 and **p < 0.003.

The LFP model-predicted excitatory and inhibitory components were calculated for each animal and the group averaged components are shown in Figure 4 across age groups (A: PA; B: YA; and C: MA). Estimated peak amplitude of the excitatory and inhibitory components, the ratio between them and the delay of inhibition with respect to excitation are shown in Figures 4D–G, respectively.

Peak amplitude of both excitatory and inhibitory components decreased as age increased. For the excitatory component, the decreasing trend was significant [*F*(2, 23) = 8.9, *p* = 0.001]. *Post-hoc* analysis showed that both the PA and the YA groups had significantly higher peak excitation than the MA group, but no significant difference was found between the PA and the YA groups. With regard to the peak amplitude of the inhibitory component, although there was a clear decrease trend with age, it did not reach statistical significance at 5% [*F*(2, 23) = 3.32, *p* = 0.054]. When comparing the ratio of inhibition to excitation across age groups using the non-parametric Kruskal Wallis test, no statistical significance was found (*p* = 0.704). For the delay of the inhibitory component with respect to the excitatory component, there was a significant increase in this delay with age [*F*(2, 23) = 8.01, *p* = 0.002]. *Post-hoc* analysis showed a significant shorter inhibitory delay for the PA group compared to that of the YA and MA groups.

## Discussion

This study used three different age groups of healthy rats to investigate the effect of aging on the somatosensory evoked field potential and to estimate the balance between neural excitation and inhibition using a mathematical model. It is important to emphasize that the age span of the rats used in the study was from pre-adolescence (1 month) to middle aged (20 months) (Quinn, 2005). Further experiments with rats older than 20 months will be needed to determine if findings from this study can be extended to the older population.

It is also worth noting that, in this study, only the granular layer LFP was used for comparison across the three age groups. As the brain weights are significantly different between groups, it is likely that cortical thicknesses are also significantly different. By aligning the LFP to the layer IV sink through CSD analysis across age groups, LFPs from other cortical layers are not aligned, making comparisons difficult for these layers. However we would expect that similar age-related decreasing trend in sensory evoked LFP amplitude would be observed across cortical depth.

### Level of Anesthesia Across Age Groups

We monitored the anesthetic level and maintained it at stage III3 during the recording period based primarily on the temporal characteristics of the extracellular field potential recordings displayed on a PC monitor. Interestingly, although we did not target a specific ISO% value for stage III3 during data collection for all subjects, the mean ISO% between the three age groups were not significantly different, shown in Figure 1E. This could be due to two confounding factors affecting the level of anesthesia in opposite directions. One is aging, the other the body weight. It has been shown that as age increases, less ISO% is required to maintain the same minimum alveolar concentration (MAC), a quantity used as the standard measure of potency for volatile anesthetic agents (Nickalls and Mapleson, 2003). On the other hand, the uptake of ISO increases with increasing weight (Pal et al., 2001). For the three age groups used in our study, increasing age was associated with increasing weight. It was thus possible that their effects on ISO concentration canceled each other, resulting in similar ISO% used to maintain stage III3 anesthesia for all three age groups.

We also noted that the heart and respiration rates for the PA group were significantly higher than the YA and MA group, but there was no significant difference in these physiological parameters between the two older groups (Figures 1C,D). Several previous studies also demonstrated similar findings in awake male Sprague-Dawley albino rats (Tomanek, 1970) and in male Fischer 344 rats (Roberts and Goldberg, 1976). As heart rates are known to be modulated by the respiration cycles (Hayano et al., 1996; Schäfer and Kratky, 2008; Sato et al., 2008), this may explain the significantly higher mean respiration rate in the YA group. These parameters alone do not indicate the depth of anesthesia. The main indicator for stage III3 anesthesia is the PSD of spontaneous LFP responses.

Figure 1G shows the three mean PSDs of spontaneous LFP for the three age groups. The peak at 3–4 Hz implies that stage III3 level of anesthesia was achieved for each group (Friedberg et al., 1999). However, a peak around 1 Hz was also observed in each PSD plot, and it was particularly prominent for the PA group. After visual inspection of the data, we found that this component was possibly due to animal respiration, which was not completely canceled by the reference electrode placed at the back of the rat’s neck. The likely cause could be the soft nose cone set up used for whisker pad stimulation while administering ISO. The head of the animal was fixed by the ear bars without the bite bar which had to be removed to allow the microflex breather to be placed. The chin and the nose of the rat were supported by the soft cone mask. This was not as secure as a bite bar, thus a small degree of head movement caused by respiration, which was not obviously observable by the experimenter, may have been recorded by the microelectrode. This movement may be more severe in younger rats as their heads were smaller and noses narrower. A possible remedy to the problem for future experiments would be to add padding between the nose cone and the chin of the rat to provide more secure support to the head of the rat.

### Aging Reduced the Temporal Dynamics of Sensory Evoked Excitatory Post-synaptic Activity

Our results demonstrated that, during healthy aging, the amplitude of the somatosensory evoked LFP in the rat barrel cortex diminishes, and its peak latency increases. Similar findings were reported in studies of the rat auditory brain stem (Alvarado et al., 2014; Parthasarathy et al., 2014, 2019), the somatosensory cortex of mice in response to forepaw and hindpaw stimulation (David-Jürgens and Dinse, 2009), and human EEG studies of visual, auditory and somatosensory cortices (Onofrj et al., 2001; Kalisch et al., 2008; Konrad-Martin et al., 2012; Price et al., 2017; Brown et al., 2019).

We further observed that the initial slope of the evoked LFP response was significantly decreased with age. Based on our previous work (Bruyns-Haylett et al., 2017), this reduction would imply an age-related slowdown of the dynamics of the excitatory post-synaptic activity. To confirm this finding in the current study, we replicated our previous experiments with two important modifications: we infused gabazine instead of bicuculline and used an isoflurane anesthetic regime instead of urethane. The initial slope of the LFP pre- and post-infusion did not change significantly, confirming that aging decreased the time constant of the excitatory post-synaptic response in the rat barrel cortex. It also implied that the initial slower temporal dynamics of the evoked LFP was not caused by enhanced inhibition in the neocortex. Despite the reduced rate of change of the evoked LFP, the onset latency did not differ significantly between the age groups, suggesting that nerve conduction velocity was maintained in rats across the age span 1–20 months. This result corroborated an earlier finding in a mice study (Walsh et al., 2014).

One possible mechanism underlying the decreased dynamics of the evoked LFP could be peripheral deafferentation (Caspary et al., 2008), resulting in reduced strength of afferent signal from thalamus to the barrel cortex. Age-related increase in sensory threshold (Kalisch et al., 2008; Alvarado et al., 2014; Stebbings et al., 2016) may also lead to reduced afferent input to the cortex. Altered synaptic dynamics in the somatosensory cortex (Mostany et al., 2013; Petralia et al., 2014) could be another contributing factor, as the size and the long-term stability of spines, as opposed to the density, were shown to reduce with age. These changes were likely to result in weaker synapses, therefore weaker neural signal transmission. An important phenomenon known to be essential for information processing of the nervous system is neural synchronization (Poulet and Petersen, 2008; Uhlhaas et al., 2009; van Wijk et al., 2012). Age-related decline in neural synchrony has been shown in studies of the auditory cortex (Harris and Dubno, 2017; Xie and Manis, 2017), and the motor cortex (Liu et al., 2017). Neural synchrony is associated with a neuron’s ability to phase lock to a stimulus, or the synchronization of release of neurotransmitters at synapses. Degradation of neural synchrony during aging may lead to reduced post-synaptic responsiveness and slower temporal dynamics of the evoked field potential. Finally it has been shown that neuronal loss is minimum during normal aging (West et al., 1994; Yankner et al., 2008; Bishop et al., 2010), thus the age-related reduction in the evoked LFP is unlikely due to reduced number of neurons in the cortex.

### Model Predicted Balance Between Neural Excitation and Inhibition Was Maintained During Healthy Aging

The amplitude and peak latency of the evoked LFP are both dependent on the amplitude of the excitatory and inhibitory post-synaptic potentials (EPSPs and IPSPs), as well as on the temporal delay of IPSP with respect to EPSP. Using a mathematical model of the LFP (Zheng et al., 2012), we decomposed the evoked LFP response into components of excitation and inhibition, and found a significant age-related decrease in the amplitude of the model-predicted excitatory component, and a consistent decreasing trend in the amplitude of the model-predicted inhibitory component (not reaching significance at *p* = 0.054). Furthermore, the lag of inhibition with respect to excitation was significantly increased with age. However, the ratio of the model-estimated peak amplitude of inhibition to that of excitation was maintained across age groups.

Although many studies have investigated age-dependent changes in sensory evoked potentials, few have addressed how evoked synaptic excitation and inhibition and their balance are altered during healthy aging. There are limited studies looking into age-related functional changes in the resting state synaptic activity (Luebke et al., 2004; Bories et al., 2013) via whole-cell patch clamp recording of action-potential-dependent spontaneous post-synaptic currents and action-potential-independent miniature post-synaptic currents (Pinheiro and Mulle, 2008). The findings were not consistent and the relationship between the resting state and the sensory evoked synaptic activities is still unclear.

A study by Alvarado et al. (2014) used a rat model of the auditory pathway to investigate molecular mechanisms underlying age-related modifications of sensory-evoked potentials. Combining auditory brainstem responses with immunohistochemical staining for the excitatory marker vesicular glutamate transporter 1 (VGLUT1), and the inhibitory marker vesicular GABA transporter (VGAT), they found a significant reduction in both VGLUT1 and VGAD in older rats compared to younger ones, as well as significant age-related reductions in the amplitudes, and increases in the latencies, of evoked auditory response waves, similar to our results presented here. It was suggested that the reduction in the excitatory and the inhibitory markers may underline the age-related changes in the evoked auditory responses observed. Explicit recordings of excitatory and inhibitory components of neural activities were not made, but their findings indicated that both may decline with age.

In the study presented here, we also did not directly measure synaptic excitation and inhibition but estimated them through a mathematical model of the LFP. A similar approach was used by Legon et al. (2016), who investigated the effect of aging on human memory networks by applying the dynamic causal model (DCM) (Friston et al., 2003) to functional magnetic resonance imaging (fMRI) data and found that older participants had enhanced functional connectivity from the prefrontal cortex (PFC) to the medial temporal cortex despite of age-related decline in recall ability. To understand the underlying mechanisms, they conducted a further experiment using transcranial magnetic stimulation (TMS) over the PFC and collected EEG data while participants performed the same memory task as in the fMRI experiment. Again DCM was used in the EEG data analysis and the estimated connection strengths between excitatory and inhibitory neural populations were used as surrogates for neural excitation (E) and inhibition (I), from which the ratio E/I was used to indicate their balance. It was found that the GABAergic connectivity strength was significantly higher in the aged group compared with the young group during the recall task, suggesting that increased inhibition was associated with decreased memory and that the E/I ratio in the PFC was reduced in the aged group.

One of the main differences between the DCM used in the above study and the LFP model used in our study is that the DCM was not designed to model the temporal dynamics of EEG or LFP signals. The model parameters were optimized by minimizing the cross spectral density of neural responses from multiple anatomical regions. For the above study, the neural signals were extracted from the gamma frequency range of the EEG data. Thus, the DCM cannot predict post-synaptic excitatory and inhibitory neural responses, nor can it predict the EEG time series. For the LFP model used in our study, the model parameters are optimized by minimizing the prediction error between the measured LFP and the model fitted LFP. As shown in Figure 4 the fit was satisfactory across all age groups.

Although mathematical models are useful tools in facilitating our understanding of biological systems, predictions from mathematical models need to be confirmed by experimental data. Thus, our model prediction that evoked excitatory and inhibitory post-synaptic activities remain balanced during normal aging needs to be further confirmed by electrophysiological experiments. One such experiment would be to obtain whole-cell patch clamp recordings of evoked excitatory and inhibitory synaptic activities across age groups (Petersen, 2017). Analysis methodologies such as spike sorting to characterize sensory evoked spiking activity of excitatory and inhibitory cells may also provide valuable insight (Reyes-Puerta et al., 2015).

### Implication on Hemodynamic Signals During Healthy Aging

As our results demonstrated age-related reduction in sensory evoked synaptic activity, it is likely that sensory evoke hemodynamic responses may follow the same trend based on research in neurovascular coupling (NVC) (Jones et al., 2004; Huneau et al., 2015). Indeed many fMRI studies have shown that the amplitude of the blood-oxygen-level-dependent (BOLD) signal decreases with age (Tekes et al., 2005; Ances et al., 2009; Ward et al., 2015). Particularly interesting is a recent human fMRI study (West et al., 2019) which showed that, under visual stimulation, the BOLD signal in the occipital region of the old adult group had significantly lower peak amplitude, longer peak latency and slower rise slope compared to the young adult group. These age-related changes of the BOLD response were remarkably similar to what we observed in our neural data (Figure 2). Additionally, the above fMRI study collected behavioral data and found no significant performance difference between the age groups. It thus hypothesized that the observed differences in the hemodynamic responses could be largely due to age-related changes in the NVC unit, rather than simply changes in neural activity.

In light of our neural data, there is a clear need for the hypothesis to be further examined. It is certainly the case that the two studies cannot be directly compared, as the neural signals presented here were obtained from a rodent study with subjects under anesthesia, while the hemodynamic signals observed in the above fMRI study came from a human study with subjects performing a cognitive task. However, it has been shown that the absence of difference in behavioral measurements of different age groups is not mirrored by sensory evoked neural data which showed age-dependent changes (Rufener et al., 2014). On the other hand, it is also known that during healthy aging, NVC is impaired because of cerebro-microvascular dysfunction (Lipecz et al., 2019). Further research using multimodal measurements coupled with mathematical models will likely provide more insight into the effect of aging on the neural and hemodynamic signals and the underlying mechanisms.

## Conclusion

Our study presented evidence that, during healthy aging in rats, somatosensory-evoked post-synaptic activity shows age-dependent reduction in its amplitude and slowdown in its temporal dynamics. Using mathematical modeling approach, we estimated the evoked excitatory and inhibitory components and found age-related amplitude reduction in both. Furthermore, the delay of inhibition with respect to excitation was found to increase with age. However, the amplitude ratio between the two components was not significantly changed across age groups. We suggest that within the age span from 1 to 20 months, synaptic excitation and inhibition in the rat barrel cortex maintain their co-tuning property to facilitate normal brain function.

## Data Availability Statement

The datasets generated for this study are available on request to the corresponding author.

## Ethics Statement

All procedures were undertaken in accordance with the 1986 Animal (Scientific Procedures) Act, under approval from the United Kingdom Home Office and approved by the Research Ethic Committee at the University of Reading, United Kingdom.

## Author Contributions

SK: conceptualization, data curation, software, formal analysis, writing – original draft, review and editing, and self-funding. YH: data curation. MB-H: data curation, methodology, and writing – review and editing. ED: writing – review and editing and project administration. YZ: conceptualization, data curation, methodology, software, formal analysis and validation, writing – original draft, review and editing, and funding acquisition.

## Conflict of Interest

The authors declare that the research was conducted in the absence of any commercial or financial relationships that could be construed as a potential conflict of interest.

## Appendix

### A Mathematical Model of the Evoked Local Field Potential

The structure of the mathematical model was given in Figure 2B of Zheng et al. (2012). We adopted the same notation here, shown in Table A1 below. As the sigmoid function was approximated by a linear operation, the entire model can be written in Laplace transfer function forms as follows:

$$P\left(s\right)=\left\{H_{eL}\left(s\right)+H_{iL}\left(s\right)e^{-\tau_{d}s}\right\}\left(U\left(s\right)-F\left(s\right)\right)$$

(A1) $$=\left\{-\frac{K_{eL}}{{\left(\tau_{eL}s+1\right)}^{2}}+\frac{K_{iL}}{{\left(1.4\tau_{eL}s+1\right)}^{2}}e^{-\tau_{d}s}\right\}\left(U\left(s\right)-F\left(s\right)\right)$$

Where

$$F\left(s\right)=\left(H_{eD}\left(s\right)+H_{iD}\left(s\right)e^{-\tau_{d}s}\right)P\left(s\right)$$

(A2) $$=\left\{-\frac{K_{eD}}{{\left(\tau_{eD}s+1\right)}^{2}}+\frac{K_{iD}}{{\left(1.4\tau_{eD}s+1\right)}^{2}}e^{-\tau_{d}s}\right\}P\left(s\right)$$

In the above equations, the variable *P* models the local field potential, and the variable *U* represents the afferent input to the cortex. There are seven model parameters, as listed in Table A1, which need to be optimized to minimize the difference between the measurement (LFP) and the model predicted output P in the least squares sense.

**TABLE A1:** Definitions of the notation used.

Notation	Definition
*K*_*eL*_	Model gain of excitatory component of LFP due to local neural population
τ_*eL*_	Time constant of excitatory component of LFP due to local neural population
*K*_*iL*_	Model gain of inhibitory component of LFP due to local neural population
τ_*d*_	Temporal delay of inhibition with respect to excitation
*K*_*eD*_	Model gain of excitatory component due to feedback from distant neural population
τ_*eD*_	Time constant of excitatory component due to feedback from distant neural population
*K*_*iD*_	Model gain of inhibitory component due to feedback from distant neural population
